# Deconstructing Racialized Experiences in Healthcare: What a Missed Opportunity for Healing Looks Like and Healthcare Resources for Children and Their Families

**DOI:** 10.31372/20200504.1109

**Published:** 2021

**Authors:** Connie K. Y. Nguyen-Truong, Shameem Rakha, Deborah U. Eti, Lisa Angelesco

**Affiliations:** a Washington State University College of Nursing in Vancouver, Washington, United States; b Washington State University College of Education in Vancouver, Washington, United States; c Washington State University College of Nursing in Spokane, Washington, United States

**Keywords:** adverse childhood experiences, Critical Race Theory, AsianCrit, families, healthcare practice, implicit bias, xenophobia, racism, Vietnamese, Vietnamese-Chinese, White fragility, equity

## Abstract

Some patients and families of color, including Asian Americans, face significant adverse stressors due to living within a White-dominant society. Xenophobia and racism can impact health. Research evidence points to early exposure to adverse childhood experiences such as racial discrimination as being detrimental and having significant short-term and long-term impact on physical and mental health. The purpose of this commentary article is to illuminate the need of patients and their families who may seek health care providers (HCPs) to express their concerns and fears when issues of xenophobia and racism arise. Patients and families need space in a healthcare setting to feel heard and understood. Anti-Asian xenophobia and racism among medically underserved Asian Americans persists and has been heightened during the COVID-19 pandemic. We describe tenets of Critical Race Theory and AsianCrit, and use this lens to understand an example actual scenario, a counter-story, of a Vietnamese mother, and her Vietnamese-Chinese American family’s experience with xenophobia and racism at a community recreation center and the subsequent communication of this experience with a HCP. We describe the impacts of these experiences of seeking healing including discontinuity of a HCP-patient-family relationship. It takes bravery for patients and families to tell their story of xenophobia and racism to a HCP. There are Asian Americans who are afraid to seek healthcare because of anti-Asian xenophobia and concerns about White fragility. Following, we highlight research evidence on implicit bias, also known as unconscious bias, as context about its persistent and widespread existence among healthcare professionals in general and the need to address this in healthcare. Implicit bias can influence care provided to a patient-family and the interactions between a HCP-patient-family. We include additional resources such as those from the National Association of Pediatric Nurse Practitioners, American Psychological Association Office on Children Youth and Families, the Office of Ethnic Minority Affairs, the Office on Socioeconomic Status, and American Academy of Pediatrics to consider in support of equity in healthcare practice of children and their families.

Our team of healthcare providers (HCPs), researchers, and educators write this commentary article to illuminate the need of patients and their families who may seek HCPs to express their concerns and fears when issues of xenophobia and racism arise; the need for a space in a healthcare setting to feel heard and understood; and consideration of theoretical frameworks and evidence including resources in support of healthcare practice. Some patients and families of color, including Asian Americans, face significant adverse stressors due to living within a White-dominant society (Thames, Irwin, Breen, & Cole, 2019). Xenophobia and racism can impact health (Le, Cha, Han, & Tseng, 2020). Research evidence points to early exposure to adverse childhood experiences such as racial discrimination as being detrimental and having significant short-term and long-term impact on physical and mental health (Anderson, Luartz, Heard-Garris, Widaman, & Chung, 2020; Conn, Szilagyi, & Forkey, 2020; National Association of Pediatric Nurse Practitioners [NAPNAP, 2020]; Trent et al., 2019; Zare et al., 2018). The NAPNAP (2020) released a statement on discrimination and child health. This statement included,

*…NAPNAP encourages all pediatric providers to continue to respond with kindness and compassion to children at this time, providing families with culturally safe care environments and effective strategies for positive coping. Anticipatory guidance at well visits should include support for children and families to recognize and respond to unfair, discriminatory and racist treatment. During this time, pediatric clinicians should be especially alert to children who exhibit signs of anxiety, depression, grief and stress*.

The American Academy of Pediatrics (AAP) underscored racism as a core social determinant of health and a driver of health inequities in their policy statement (Trent et al., 2019).

In this commentary article, we begin by describing critical race theory (CRT) and AsianCrit that has roots in CRT. We use these theoretical frameworks to help us understand an example actual scenario (using pseudonyms) involving Lan and her family’s experience with xenophobia and racism at a community recreation center and the subsequent communication of this experience with a HCP. We discuss the actual scenario as an example of a counter-story using the lens of CRT and AsianCrit to achieve an in-depth description of the impacts of these experiences of seeking healing. It takes bravery for patients and families to tell their story of xenophobia and racism to a HCP. Le et al. (2020) assert that there are Asian Americans who are afraid to seek healthcare because of anti-Asian xenophobia. Following, we highlight research evidence on implicit bias, also known as unconscious bias, as context about its persistent and widespread existence among healthcare professionals in general and the need to address this in healthcare. Implicit bias can influence care provided to a patient-family and the interactions between a HCP-patient-family. We include additional resources to consider in support of equity in healthcare practice of children and their families.

## Theoretical Frameworks: CRT and AsianCrit

CRT and AsianCrit frameworks offer tenets as a way of thinking about situations critically and through the lens of race.

### Tenets of CRT

CRT has its roots in legal studies. Developed in the 1970s as a response to the unwillingness of those in the legal field to respond to the role of race and racism in the legal system (Delgado & Stefancic, 2013), CRT centers the experiences of people of color and works from a series of tenets. Critical race scholars have identified the core tenets of CRT in diverse ways. The following are the tenets put forth by Delgado and Stefancic (2013):

1. There is no biological foundation for racial categories, as race is a socially constructed phenomenon.
2. Racism is normal and pervasive within American society.
3. Rewriting and reanalyzing history is a way of exposing the ways that race and racism permeate society and function to oppress people of color.
4. Different racial groups are racialized in disparate ways.
5. White people who hold decision-making power will only support laws, policies, or programs that benefit White people.
6. There are no essential experiences or attributes that defines any group of people.
7. Race intersects with class, gender, sexuality, ability, and other identities to shape systemic forms of oppression and individual experiences.
8. Oppressed people have stories that can constitute valuable knowledge and can be used to counter dominant narratives.

CRT scholars argue that the traditional claims of objectivity, meritocracy, colorblindness, race neutrality, and equal opportunity act “as a camouflage for the self-interest, power, and privilege of dominant groups in U.S. society” (Solórzano & Yosso, 2002, p. 26).

Solórzano and Yosso (2002) argue that CRT advances, foregrounds, and accounts for the role of race and racism in society. This theory is interdisciplinary, drawing on the fields of sociology, women’s studies, law, humanities, ethnic studies, and history. The goal of CRT is to eliminate racism and other forms of subordination (e.g., such as those based on gender, language, citizenship, and social class) by exposing and understanding the way they work in society (Liu, 2009). CRT challenges normative White values and furthers a more complex understanding of racial dynamics by utilizing storytelling and personal narratives to expose an alternative perspective or what critical race scholars call, “counter-story” or, “counternarrative.” According to Solórzano and Yosso (2002), “The counter-story is a tool for exposing, analyzing, and challenging the majoritarian stories of racial privilege” (p. 32). According to these authors, by using scenarios, personal narratives, and family histories the researcher and author is able to explain the racialized, gendered, and classed experiences of people of color. Though developed to speak to the condition of Black people in particular, this theory has since been applied to other minoritized groups. One such application has been used to help understand the experiences of Asian people in America, and it is called AsianCrit.

### Tenets of AsianCrit

AsianCrit can be viewed as a conceptual lens to study how racial oppression shapes the lives of Asian American people and communities. AsianCrit, like CRT, has a set of interconnected tenets that build upon the tenets of CRT and incorporates additional knowledge of Asian American racial realities (Museus & Iftikar, 2013).

1. Racism is pervasive in American Society, and America racializes Asian Americans in a distinct way. This tenet focuses attention on the fact that society aggregates all Asian Americans into a monolithic group and racializes them as overachieving model minorities and perpetual foreigners. This tenet, labeled *Asianization*, recognizes the ways in which Asian American men are emasculated and Asian American women are hypersexualized and seen as submissive. It also recognizes the ways in which categorizing Asians as model minorities creates a racial hierarchy, and therefore pits people of different minoritized groups against one another.
2. *Transnational Contexts* highlights the importance of historical and contemporary national and international contexts for Asian Americans. Museus and Iftikar (2013) offer this example: “U.S. military intervention in Southeast Asia contributed to the displacement of many Cambodian, Hmong, Laotian, and Vietnamese refugees” (p. 25).
3. *(Re)Constructive History* underscores the importance of “(re)constructing a historical Asian American narrative” (Museus & Iftikar, 2013). This tenet emphasizes reanalysis of history to expose racism towards Asian Americans and to allow for the inclusion of the voices and contributions of Asian Americans to the United States.
4. This tenet assumes that race is a socially constructed phenomenon. *Strategic (Anti)Essentialism* recognizes that dominant oppressive forces impact the ways in which Asian Americans are racially categorized and racialized in society. Strategic (Anti)Essentialism suggests that effective research and activism “should generate an understanding of Asian American communities as a whole and build on the possibilities for unity provided by the larger racial category while recognizing and developing intricate knowledge of the diversity and complexity that exists within these populations” (Museus & Iftikar, 2013, p. 26).
5. *Intersectionality* in AsianCrit is based on the notion that racism and other forms of oppression intersect to mutually shape the living conditions for Asian Americans (Crenshaw, 2017). This tenet emphasizes the need for application of intersectionality to help facilitate deeper and more “complex multilayered analysis of the ways in which social structures, political processes, and identities intersect to create certain conditions, realities, and experiences than what already exists” (Museus & Iftikar, 2013, p. 27).
6. *Story, Theory, and Praxis* emphasizes the idea that counter-stories, theoretical work, and practice are intertwined elements in the analysis of Asian American experiences. AsianCrit analyses stories of Asian Americans for positive transformative practices.
7. The last tenet, *commitment to social justice*, is the notion that AsianCrit is dedicated to research, unearth, and end all forms of oppression (Matsuda, 1991).

### CRT and AsianCrit Use Counter-Stories to Help Understand Incidents

Critical race theory and AsianCrit use counter-stories to help understand incidents that may be perceived as being race-neutral. In Box 1, Lan tells her and her family’s own story.


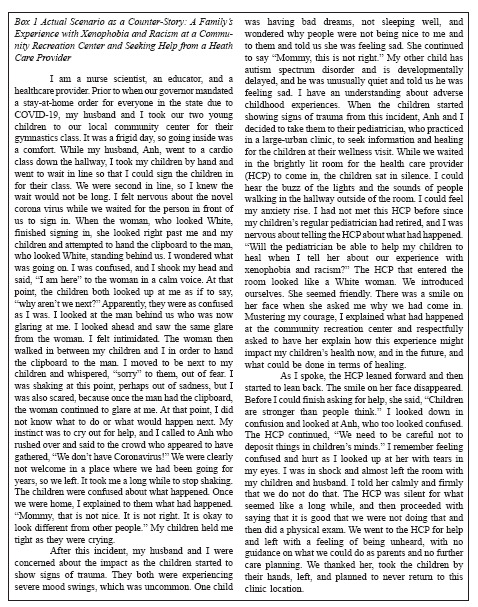


Next, we use the lens of CRT and AsianCrit to discuss this scenario from the perspective of those receiving care. What occurred at the community recreation center was not race neutral. Lan who is Vietnamese and her children who are Vietnamese-Chinese, experienced xenophobia and racism for being Asian. In AsianCrit, part of the tenet of Asianization is the unwarranted aggregating of all Asians. That is what appeared to have happened in this scenario. The woman, possibly fearing COVID-19, or, as it was described by some in the media at that time, “the China Virus,” did not want to hand the clipboard to someone who appeared to be Asian.

Our discussion is not on the incident at the community recreation center but what occurred in the subsequent healthcare visit, through the parents’ perspective about a missed opportunity for healing that underscores a potential reason for discontinuity in healthcare for people experiencing xenophobia and racism. When discussing such incidents, it is important to note that we are not meaning to suggest that the HCP purposefully or intentionally enacted racist acts, but rather, we want to use these frameworks to illuminate how HCPs actions may be interpreted by those who are minoritized within the healthcare system, even when the best of intention is employed. Psychologists suggest that some of our actions are not predicated upon our stated or conscious beliefs or principles, but rather, some of how we act is unconscious and is based on our biases. According to Greenwald and Krieger (2006), “implicit biases are discriminatory biases based on implicit attitudes or implicit stereotypes” (p. 951). Implicit or unconscious bias has been described as “habits of the mind learned over time” and stored in memory (Burgess, Beach, & Saha, 2017, p. 372). We later expand upon and highlight research evidence on the existence of implicit bias among healthcare professionals in general.

In this scenario, Lan was afraid of the potential impacts the racist incident the family experienced at the community recreation center may have on her children, therefore, she and her husband brought their concerns to the children’s pediatrician, seeking information and healing. Lan shared having to “muster courage” to talk about this situation with the HCP. She was, like many people of color, concerned whether talking about a racist incident may cause the HCP to feel uncomfortable, and possibly act in a way that DiAngelo (2011) defines as being a hallmark of White fragility. In *White Fragility* (2011), DiAngelo discusses ways White people respond to racist incidents, and one such response is for people to shut down or to react by suggesting the incident was not as bad as it seemed. When Lan described what she and her children had experienced at the recreation center, the HCP asserted, “children are stronger than people think.” Though it may be true that people underestimate the strength of children, in this circumstance, where the children experienced and witnessed an act of xenophobia and racism against their mother, this incident may have caused what Comas-Díaz, Hall, and Neville (2019) call “racial trauma.” Similar to post traumatic stress disorder, racial trauma involves injuries due to exposure to race-based stress. Race-based stressors include threats of harm or injury, humiliating and shaming events, and witnessing harm to other people of color due to racism (Carter, 2007). Though researchers recognize that African American people experience race-based stress at a higher rate (Chou, Asnaani, & Hofmann, 2012), Asian Americans, along with other minoritized groups, also significantly suffer from race-based stress (Comas-Díaz et al., 2019). This family experienced a race-based stress, and they went to the HCP for help. However, they reported not having received further guidance on what they could do as parents nor did they receive further care planning for their children. These children are Asian, living amidst the COVID-19 pandemic that is leading to significantly increased incidents of anti-Asian xenophobia and racism (Cheah et al., 2020; Choi & Kulkami, 2020). Xenophobia and racism towards Asians and Asian Americans, such as that shared in this scenario, demonstrate the need for applying AsianCrit and centering race and racism when addressing issues that impact Asian Americans (Liu, 2009). Decentering race can be perceived as dismissing the xenophobia and racism faced by this family. By saying “children are stronger than you think,” the parents perceived that their concerns for their children’s health and wellbeing was rendered null.

Ford and Airhihenbuwa (2010) assert that because racism is ubiquitous, White people often cannot see or understand the racism people of color face. Parents of color (includes caregivers/guardians; hereafter referred to as parents) do not have the privilege of not teaching their children about racism because it affects their lives. For example, the reporting center, STOP AAPI HATE received more than 2,373 reported incidents of xenophobia, racism, hate speech, discrimination, and physical attacks against Asians and Asian Americans from March to July 2020 (Human Rights Watch, 2020). Anti-Asian xenophobia and racism among medically underserved Asian Americans persists and has been heightened during the COVID-19 pandemic.

What the parents needed in this situation was to have an opportunity to be heard and understood. They needed information that would help them make the best-informed healthcare decisions for their children moving forward. Being heard and understood can lead to healing. Murray and McCrone (2014) found that the interaction between a HCP and patient-parent, the way they relate to one another, and how well they communicate together, can affect the quality of care the patient receives and the outcomes. In this situation, there appeared to be a communication breakdown that led to a discontinuity of care.

Instead of information, the parents were told they may have hurt their children by talking to them about the incident. This produced feelings of being invalidated. Instead of participating in a shared decision-making in the care of children, the HCP came across, to the parents, as offering a cautionary message. CRT and AsianCrit allow centering the story of Lan and her family in their own voices. We present their story here as way to help understand how HCP’s actions can be perceived. By applying these theoretical frameworks to this example scenario, we illuminate that xenophobia and racism have an impact on adults and children alike. It also helps to bring out personal implicit biases that may have an impact on the ability to hear and understand people’s stories. Finally, this perspective helps to understand that regardless of race, people come to HCPs for help and understanding. Next, we will discuss implicit bias in general.

## Implicit Bias

We highlight research evidence on implicit bias as context about its existence among healthcare professionals in general. Holroyd (2015) described unconscious biases as having the potential to distort a person’s judgment to the extent that they may not feel responsible for their behavior. Usually, these implicit associations do not align with the values and beliefs a person claims to have but can be unintentionally triggered to the extent that a person’s behaviors and judgments are significantly altered. An affected person may have “nonprejudiced intentions,” but their actions reveal an influence from personal experiences, and deeply ingrained cultural values that have been adopted and that are difficult to control (Burgess et al., 2017, p. 372). Implicit bias can influence the way healthcare is provided to a patient-family and in the interactions between HCP and patient-family.

Research evidence points to the persistence of and widespread nature of implicit bias, and this may be maintained in numerous ways. Implicit bias can unconsciously shape a person’s behavior and potentially lead to differences in the clinical decision making for patients on account of their race, ethnicity, or gender (Chapman, Kaatz, & Carnes, 2013). Racism and behaviors based on implicit bias in the healthcare environment can have a profound impact on the wellbeing of children and adolescents (Trent et al., 2019). These implicit behaviors, regardless of whether they are institutional or personally mediated, can impact health equity, mental health, and overall wellbeing of children and adolescents (Trent et al., 2019). Key developmental milestones can be affected even when a racist experience involves the child merely observing as a bystander (Trent et al., 2019). While certain stressors can spur on the initiation of implicit bias in the healthcare environment, Holroyd (2015) argued that this is not an excuse to discriminate against members of certain racial groups. Sometimes, individuals may not be aware that their actions are suggestive of implicit bias, however not having awareness does not absolve them from accountability (Holroyd, 2015). HCPs play a crucial role in addressing implicit bias.

Implicit bias needs to be addressed in the healthcare setting. HCPs are encouraged to confront their biases and engage in intentional behavioral change. While the Implicit Association Test (IAT) encourages engagement in self-reflection exercises to improve a person’s self-awareness, Burgess et al. (2017) contend that this intervention presents a potential drawback. Rather than aim for “procedural knowledge” that focuses on the “how,” the IAT concentrates on “declarative knowledge” or the act of “knowing” something (p. 374). By taking the IAT, people can become aware of their implicit biases, but Burgess et al. argue that there is a difference between knowing and practicing thinking and acting in a different way. Procedural knowledge allows a person to be both aware of the existence of implicit bias and to examine their feelings and act on them daily (Burgess et al., 2017). The emphasis here is on the repeated practice of a skill to the extent that it is enhanced over time.

Though having knowledge of one’s own biases is not enough to inhibit the effect of our implicit biases, several researchers have suggested ways such bias may be mitigated. Chapman et al. (2013) explained that making a deliberate effort to focus on specific information about an individual and envisioning their viewpoints are more effective ways to mitigate the impact of implicit bias. Research evidence suggests showing compassion, empathy, and mutual respect, can help decrease the instinctive stereotyping of suffering patients and improve the patient–provider relationship (Boscardin, 2015; Burgess et al., 2017; FitzGerald & Hurst, 2017; Trent et al., 2019). Burgess et al. (2017) highlighted that the use of meditation and mindfulness practices can decrease the likelihood that any biases will be activated in a person’s mind in the first place. Trent et al. (2019) stated that health care professionals who care for the pediatric population are also charged with proactively engaging in activities that reduce racism and stereotyping. Next, we will discuss additional resources for consideration in support of healthcare practice with children and their families.

## Additional Resources for Consideration in Healthcare Practice

We describe the following resources that can also support practice in the care of children and families. NAPNAP (2020) recommended *A Practical Guide to Child and Adolescent Mental Health Screening, Early Intervention, and Health Promotion, 2nd Edition* that includes guidance for parents and children coping with stress, anxiety, uncertain events, and loss (Bernadette, 2013). Parents need guidance on how to prepare for and engage in conversations with their children regarding race and ethnicity. NAPNAP recommended the American Psychological Association Office on Children Youth and Families (APAOCYF), the Office of Ethnic Minority Affairs (OEMA), and the Office on Socioeconomic Status (OSS; n. d.) on strategies regarding racial ethnic socialization and the Substance Abuse and Mental Health Services Administration (U. S. Department of Health & Human Services, 2020) on family/caregiver guidance during times of traumatic stress as resources. Racial ethnic socialization, a process through which children learn about race and ethnicity, includes learning about how to communicate with others about race and ethnicity and developing skills to successfully deal with topics that may arise around race and ethnicity (APAOCYF, OEMA, OSS, n.d.). Intentional guidance in the messaging may help in parent(s)-child(ren) communication. Messages include “Things SAID and UNSAID,” “Things DONE and NOT DONE,” and “emotional reactions” (APAOCYF et al., n.d.). They further assert to address conversations about race and ethnicity rather than avoiding, as when done, children’s self-esteem is higher, and children can recognize and respond more appropriately to racially charged situations. Parents are to be encouraged to stay supportive, keep communication open, and revisit the discussion on race and ethnicity with their children (APAOCYF et al., n.d.). See Box 2 for an example in how parents can prepare for and engage in racial ethnic socialization with elementary-aged children.


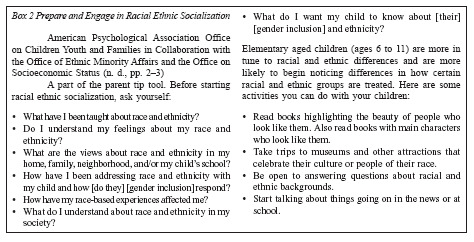


HCPs have crucial roles as caregivers, advocates, and community leaders in recognizing and addressing the detrimental effects that xenophobia and racism have on the physical and psychological health of children, adolescents, and their families. It is vital that HCPs take action to provide support and guidance that may improve health outcomes, as well as the general healthcare experience, when it is apparent in their practice that issues of structural or personally experienced racism are affecting the health of patients and their families.

Strategies for HCPs to consider integrating into practice:

- Continually examine and acknowledge personal biases and take steps to eliminate them. Mitigate the effects that these biases may have on clinical decision-making.
- Learn about the racism your patients experience by deeply listening to their personal stories and narratives and integrating this knowledge into practice to inform clinical care decisions.
- Assess patients for racial stressors such as discriminatory treatment or race related bullying as well as social determinants of health such as neighborhood safety, housing inequity, and financial strain that may contribute to or exacerbate mental health conditions such as post-traumatic stress disorder, racial trauma, anxiety, or depression (Trent et al., 2019).
- Create a medical home for patients that is culturally safe, where all areas of practice are sensitive to the effects of racism on the health and well-being of patients. Provide a medical home that ensures all people are welcome, will be treated with respect, and will be provided with quality, equitable care (Trent et al., 2019).
- Ensure that all clinical and administrative staff are trained in providing culturally competent and responsive and linguistically appropriate care and services.

## Conclusion

The tenets of CRT and AsianCrit frameworks may be helpful when working with people of color: racism is ubiquitous and real; that not all people of color or people within the same racial group are alike; that people’s stories matter and can be used to help illuminate what might be going on; that everyone deserves to be heard, but many experience being silenced; and that people of color tend to be underserved including in the healthcare setting. Therefore, standards in care must be taken to recognize biases. Through this example actual scenario as a counter-story, we illuminated the crucial need for HCPs to provide support for their patients who have experienced xenophobia and racism. We discussed this family’s experience with discontinuity of care and in doing so have provided and achieved an in-depth lens with which to view people’s stories and narratives and have provided a set of resources and practices HCPs can engage in with their patients-families that can help to improve equity in health care for people of color.

## Acknowledgments

The authors are appreciative of the anonymous peer reviewers for assistance.

## Declaration of Conflicting Interests

The authors declared no conflicts of interest with respect to the work, authorship, and/or publication of this article.

## Funding

Dr. Connie K. Y. Nguyen-Truong received the Washington State University Vancouver Nursing Excellence in Research Award that funded in part the scholarly work.
